# The impact of facility characteristics on the use of antipsychotic medications in nursing homes: a cross-sectional study

**DOI:** 10.1186/s13584-016-0070-y

**Published:** 2016-03-16

**Authors:** Dvora Frankenthal, Gisele Zandman-Goddard, Yael Ben-Muvhar, Bat Sheva Porat-Katz

**Affiliations:** Braun School of Public Health and Community Medicine, Hebrew University-Hadassah, Jerusalem, Israel; Department of Medicine ‘C’, Wolfson Medical Centre, Holon, Israel; Tel-Aviv District Health Bureau, Ministry of Health, Tel-Aviv, Israel; Faculty of Agricultural, Food and Environmental Quality Sciences, The Hebrew University, Rehovot, Israel

**Keywords:** Israel, Geriatrics, Nursing homes, Antipsychotic medications, Elderly, Facility characteristics

## Abstract

**Background:**

Antipsychotic medications (APMs) are commonly prescribed in nursing homes (NHs) and their excessive use raises concerns about the quality of care. They are often seen as “chemical restraints”, and were shown to increase morbidity and mortality risks in NH residents. The objective of this study was to investigate the variability in prevalence in APM use in a sample of Israeli NHs and to examine the effect of facility characteristics on the use of APMs.

**Methods:**

A retrospective cross-sectional study was conducted in 2011 using data which were collected in a sample of NHs in the Tel Aviv district during the annual certification process. Prevalence of APMs was determined on the basis of all residents using antipsychotics on a regular basis. The association between facility characteristics and APM use was assessed by multivariate analysis.

**Results:**

Forty-four NHs providing care for 2372 residents were investigated. The prevalence of APM use varied between facilities from 14.8 to 70.6 %, with an overall prevalence of 37.3 %. Multiple linear regression analysis revealed that greater use of APMs was associated with for-profit facilities, facilities in which most of the residents were self-pay, the presence of a “mentally frail” unit, a medical director non-specialized in geriatrics, shortage of social workers and occupational therapists, presence of unsafe/non-fitting equipment or self-aids (e.g., unsafe bath/toilet seats, unsuitable height of tables) and shortage of recreational activities.

**Conclusions:**

A wide variation in APM use was recorded in NHs in the Tel Aviv district. This variation was associated with facility characteristics that undermine quality of care. Application of APM use as a measure of quality in NHs and publicizing their utilization may decrease their overall use.

## Background

The widespread use of antipsychotic medications (APMs) in nursing homes (NHs) has been a matter of concern for many years [1]. APMs are often seen as “chemical restraints”, and they have been shown to increase morbidity and mortality risks in NH residents [2]. Newer atypical antipsychotic therapies (e.g., risperidone, quetiapine, olanzapine) were introduced in the 1990s, and they were thought to be safer than the early conventional antipsychotic therapy (e.g., haloperidol, zuclopenthixol, perphenazine, thioridazine). However, evidence subsequently showed that the use of atypical agents was associated with serious adverse events, including falls, abnormal gait, stroke and even death [3]. Despite uncertainties about the benefits and risks of APMs in the elderly, their prevalence remains high in NHs. A recent study reported an overall 32.8 % prevalence of APMs use in a voluntary sample of NH residents with dementia in 7 European countries and Israel. APM use differed by country, ranging from 60 % in 10 NHs in the Czech Republic to 18 % in 7 NHs in Israel [4]. Additionally, US data indicated that nearly one-third of elderly NH residents with dementia received APMs, mainly atypical agents [5].

The licensed indications for APMs in Israel are based on their FDA approval which was received for demonstrated efficacy in schizophrenia, psychotic disorders and, more recently, for bipolar disorder and unresolved severe depression. Although generally not approved for this use, APMs are prescribed in NHs mainly for off-label indications e.g., to manage dementia associated behavioral disturbances [6]. Specifically, behavioral symptoms include aggression, wandering and screaming, and they constitute a major cause of staff distress [7]. The potential misuse of APMs is an ongoing quality concern in NHs, particularly because these medications simply sedate residents who exhibit disturbing behaviors or resist care [2]. The boredom and isolation that result from inactivity and the monotony of the NH environment may lead to many of the behavioral symptoms exhibited by people with dementia [7]. Furthermore, needs such as those pertaining to relief of discomfort and pain have also been shown to be associated with difficult behaviors [8].

Some studies have suggested that there was an institutional “culture” for prescribing APMs. One study showed that residents entering NHs with the highest facility-level antipsychotic rates were 1.37 times more likely to receive APMs compared to those entering the lowest prescribing rate NHs [9]. Another study found that residents in facilities with high APM prescribing rates were about 3 times more likely to be prescribed them than residents in facilities with low prescribing rates, irrespective of their clinical indications [3]. Given the serious events associated with the use of antipsychotic therapy, it is important to explore the relationship between the NH environment and the prescription of APMs.

The aim of this study was to investigate the variability in prevalence in APM use in a sample of Israeli NHs and examine the effect of facility characteristics on the use of APMs.

## Methods

### Data source

This retrospective cross-sectional study used two merged data sources. One is the computerized geriatric department data system (AGAM), which provides facility level information for all certified NHs in Israel. A multidisciplinary team of national inspectors, which is comprised of experts from the fields of medicine, nursing, physiotherapy, occupational therapy, nutrition and pharmacology, routinely collects data on each geriatric facility as part of the annual certification process. They retrieve data, which include structure and process indicators, on descriptive characteristics (i.e., ownership, occupancy), levels of the staff members (i.e., qualifications, expertise), and they evaluate multidisciplinary working procedures in order to measure the quality of care in the facility. The other source involves the prescribing of APMs (an outcome indicator), which was assessed only during 2011 by the study pharmacist in a sample of NH residents in the Tel Aviv district during the annual certification process. The Ministry of Health supervises the prolonged hospitalization for the nursing and mentally frail elderly. NHs comprise different types of wards and residents are stratified by their level of disability. Data were collected for residents with and without cognitive impairment who were dependent in carrying out their activities of daily living (nursing wards), and for residents who were primarily cognitively impaired but were able to walk independently (mentally frail units).

### Study sample

The Tel Aviv district is composed of 10 cities which are located in central Israel. Forty-four of the 60 geriatric facilities within the district were randomly selected (about one facility a week) for assessment of APM prescribing policies in 2011. We used the data which were collected in those facilities for this study. The study protocol was reviewed and approved by the E. Wolfson Hospital’s institutional ethical review board which waived informed consent.

### Prescribing protocol

APMs are prescribed by physicians employed in NHs. The prevalence of APMs was determined on the basis of all residents ingesting them on a regular basis on the day of the inspection. Residents were classified as not taking any APMs, taking one APM (either a conventional or an atypical agent) or taking more than one APM.

### Facility characteristics

The selected facility predictors that were likely to have an impact on APM use included: ownership (profit or non-profit), source of payment by residents (most of the residents in the facility were self-pay or not self-pay [the latter payment to the facility being subsidized by the Ministry of Health]), number of beds, shortage of staff members (fewer working hours per month for each discipline than the professional standard set by the Ministry of Health), specialization of the medical director (geriatrician/non-geriatrician), the presence of a “mentally frail” unit (not all NHs have these units and they can be present in profit or non-profit facilities), the existence of diversified and adequate recreational activities (a variety of activities existed and activities were carried out daily), treatment by an occupational therapist (treatment was categorized into “absent”, “partial” and “available” while “absent” was defined as no treatment due to the lack of an occupational therapist and “available” was defined as the availability of treatment to all residents who need it), pain assessment (pain being measured at least once weekly among all residents), suitable outdoor environment (i.e., enough space for strolling, access to a garden or open area), safe and well-fitting self-aid equipment (e.g., safe bath/toilet seats, height of tables fitted to residents’ needs) and the use of minimum physical restraints (a prevalence of ≤10 % of patients on the day of inspection).

The selected facility characteristics for the present study were measured by the multidisciplinary team of national inspectors during the annual certification process, each one in his/her field of expertise and were derived for this study as yes/no present/absent replies.

### Statistical analysis

Statistical analysis was performed using the Statistical Package of Social Sciences (SPSS) version 14.0 (Chicago, IL, USA). Multivariate analysis using a backward linear regression was applied to examine the association between independent risk factors and the use of APMs. The following variables were entered in the linear regression model: facility characteristics (number of beds, profit/non-profit facilities, most of the residents in the facility were self-pay/not self-pay patients), specialization of the medical director (geriatrician/non-geriatrician), shortage of medical staff (physicians, nurses, occupational therapists, physiotherapists, dieticians and social workers), presence of a “mentally frail” unit, recreational activities, safe and well-fitting environment/equipment, pain assessment, treatment by an occupational therapist and minimum use of physical restraints. Significance was set at *P* < 0.05.

## Results

### Characteristics of the geriatric facilities

A total of 44 geriatric facilities had been randomly selected for inspection during the annual certification process from January 1, 2011 to January 1, 2012. Table 1 provides a descriptive overview of the 44 facilities (2372 residents) included in the analysis. Most (65.9 %) of them were for profit, and the medical director was specialized in geriatrics in 17 (38.6 %). The most prominent shortage of medical staff was that of occupational therapists (59.1 %), followed by a shortage of dieticians (43.2 %) and nurses (38.6 %). Data on the quality of recreational activities in the facility showed that it was neither diversified nor adequate in 21 (47.7 %) facilities. The physical environment was not adequate (not enough space for strolling or no access to a garden or open space) in 15 (34.1 %) facilities, and the equipment used by residents was not safe/well-fitting in 19 (43.2 %).

**Table 1:** Characteristics of the facilities (*N* = 44)

Facility characteristics	No of facilities %
Number of nursing beds in the facility	
<60	27 (61.4 %)
60–150	14 (31.8 %)
>150	3 (6.8 %)
Medical director has specialization in geriatrics	17 (38.6 %)
Shortage in personnel	
Physician	7 (15.9 %)
Nurse (with and without an academic degree)	17 (38.6 %)
Social Worker	8 (18.2 %)
Physiotherapist	9 (20.5 %)
Occupational Therapist	26 (59.1 %)
Dietician	19 (43.2 %)
For-profit facility	29 (65.9 %)
Facilities in which most of the residents are self-pay patients	22 (50 %)
Has a "mentally frail" unit	8 (18.2 %)
Non-suitable environment (i.e., not enough space for strolling, no access to a garden or open area)	15 (34.1 %)
Unsafe/non-fitting self-aid equipment (e.g., unsafe bath/toilet seats, unsuitable height of tables)	19 (43.2 %)
Recreational activities are not diversified and not adequate	21 (47.7 %)
Treatment by occupational therapist	
Absent	11 (25 %)
Partial	22 (50 %)
Available	11 (25 %)
Use of minimum physical restraint (≤10 % of patients)	17 (38.6 %)
Pain is not routinely measured among all residents	19 (43.2 %)

### Variation in the use of antipsychotic therapy

The prevalence of APM use varied between facilities from 14.8 to 70.6 %, with an overall prevalence of 37.3 %. Of all the residents who were prescribed an APM, an average of 90.6 % received only one APM and an average of 58.15 % were prescribed atypical APMs. Risperidone was the most common atypical APM (30.7 %), followed by quetiapine (16.7 %) and olanzapine (5.4 %).

Multiple linear regression analysis (Table 2) revealed that there was more prescribing of APMs in for-profit facilities (β = −0.37, *P* = 0.008), when the medical director of the facility was not a geriatrician (β = 0.46, *P* = 0.001), when there was a “mentally frail” unit (β = 0.47, *P* = 0.001), when most of the residents were self-pay (β = -0.64, *P* = 0.001), and when there were shortages of social workers (β = 0.31, *P* = 0.019), occupational therapists (β = 0.33, *P* = 0.017), safe/well-fitting equipment (β = 0.31, *P* = 0.037), and recreational activities (β = 0.32, *P* = 0.018). The number of nursing beds, the shortage of physicians, nurses, dieticians and physiotherapists, the use of physical restraint, treatment by an occupational therapist, provision of a suitable environment and routine measurement of pain were not predictors for the prescription of APMs.

**Table 2:** Predictors of antipsychotic medication use

	Unstandardized coefficients B	Standardized coefficients β	t	Sig
Medical director has no specialization in geriatrics	11.74	0.46	3.86	0.001
Has a "mentally frail" unit	15.51	0.47	3.65	0.001
Most residents are self-pay	−15.62	−0.64	−3.68	0.001
For-profit facility	−9.80	−0.37	−2.83	0.008
Shortage of social workers	9.76	0.31	2.48	0.019
Shortage of occupational therapists	8.29	0.33	2.52	0.017
^a^Recreational activities are not diversified and not adequate	18.60	0.32	2.49	0.018
^b^Unsafe/non-fitting self-aid equipment	7.70	0.31	2.18	0.037

*Sig* significance

^a^Recreational activities were measured by an inspector who is an occupational therapist

^b^Aids were judged as being safe and well-fitting according to an inspector who is a nurse

## Discussion

The findings of the current study demonstrate that the use of antipsychotic therapy varies considerably across NHs in the Tel Aviv district, and that this variability is associated with facility characteristics. The prevalence of APM use ranged between facilities from 14.7 to 70.6 %, with an overall of 37.3 % and is comparable with results from other studies [1, 3–5, 10, 11]. Hughes et al. reported a wide variability (ranging from 0 to 75 %) in the use of APMs in 14,631 US long-term care facilities [12]. Those authors showed that facility and resident characteristics were associated with the use of APMs, although the extent to which these factors explained this variability differed according to the financial incentives of the facility (for-profit, nonprofit or government) [12]. Another study carried out one decade later on a random sample of 1257 US NHs reported that facility-level APM prescribing rates ranged from 0 to 100 % [9]. That study showed that patients diagnosed with schizophrenia, bipolar disorder or aggressive behavioral symptoms of dementia accounted for only a small proportion of APM use, and the authors concluded that a prescribing culture exists in NHs. These findings demonstrate the persistent variability of APMs in NHs in the US which have not changed appreciably since first reported in 2000. In The Netherlands, a wide variation in APM use was demonstrated in a voluntary sample of 20 long-term care facilities ranging from 5 to 52 %, with an overall prevalence of 31 % [1]. That study showed that facilities with a high prevalence of APM use were often large, situated in urban communities and scored below average on staffing, personal care and recreational activities [1]. In Ontario, Canada, antipsychotic prescribing in 485 provincially regulated NHs ranged from 20.9 to 44.3 % [3]. The variation in APM use in Canada was not adequately accounted for by measured differences in the characteristics of the residents, and it was concluded that antipsychotic therapy was not based on clinical indications but rather on the NH environment, with some environments being more permissive than others about APM use [3]. The authors did not determine what it was about the NH that influenced prescribing.

The variability in APM use in the surveyed Israeli NHs was associated with facility characteristics. The present study demonstrated an association between non-profit facilities and lower prevalence of antipsychotic use. This is consistent with the findings in other studies, and it reflects the underlying assumption that higher-quality care may be a more important goal than maximizing profit in non-profit facilities [12–14]. Aaronson et al. [15] observed that non-profit facilities provided significantly higher quality of care to residents compared with for-profit NHs, as demonstrated by better health outcomes among residents with higher risk for adverse outcomes. Furthermore, Hughes et al. [12] reported an inappropriate use of APMs as chemical restraints in NH environments in which there was more demand upon increasing profits.

The current study demonstrated that the source of payment was also associated with APM use. There was higher APM use in facilities with more self-pay residents. The Ministry of Health supervises all certified NHs regardless of the payer source and non-self-pay residents can go either to non-profit or for-profit facilities. The Ministry of Health’s reimbursement to facilities for non-self-pay residents may result in the delivery of better care to such patients as reflected by lower APM use. We believe that credit for this finding should go to the constant supervision by the Ministry of Health on the services supplied to those residents. Further investigation into this assumption is warranted.

Specialization in geriatrics by the medical director was associated with a reduced use of APMs. The medical director of a NH is in charge of the supervision of all medical activity including prescribing decisions. Azerramai et al. reported that APM use was significantly lower in NHs where patients received treatment from a general practitioner with additional training in gerontology [16]. These findings support the likelihood that perceptions shared by physicians working within a NH may be expressed as facility-level preferences for certain therapeutic regimens [9]. We suggest that a physician with a specialization in geriatrics may promote non-pharmacological interventions as first-line treatments for behavioral problems of dementia before the initiation of APMs more than other physicians.

Shortages of occupational therapists and social workers were found to be associated with a greater use of APMs. The lack of sufficient and qualified staff was reported as a barrier to the success of non-pharmacological interventions, thereby increasing the demand for APMs [1, 17]. The use of medications as chemical restraints can serve as substitutes for staff members. APM use was reportedly less prevalent in facilities that were well run and where staff levels were appropriate with resident needs [12].

As could be expected, more APMs were used in facilities with a “mentally frail” unit. More of those residents were diagnosed with dementia, and the prevalence of behavioral problems associated with dementia was higher in those facilities.

The level of recreational activity in a facility was also associated with APM use. Kleijer et al. found a strong association between low satisfaction with the provided recreational activities and facility-level APM use [1]. The use of recreational activity programs can improve the residents’ quality of life by reducing the boredom and isolation that may precipitate behavioral problems, and thus decrease the demand for APM use [18, 19]. Most agitated behaviors are manifestations of unmet needs [8]. According to those authors, the most common of those needs are for social and physical stimulation, both of which are lacking because of the effects of dementia, sensory deficits and the monotony of the NH environment [8]. Recreational programs promote quality of life by providing an appropriate level of stimulation by means of meaningful activities [7].

Poorly fitting equipment, such as unadapted wheelchairs, dining/work tables or old non-fitting toilet/bathing seats were associated with a greater use of APMs.

Environmental interventions which meet both the comfort and the mobility needs of residents were suggested for promoting the residents’ highest level of functioning [8].

This study has several limitations. First, its cross-sectional design precluded the determination of a causal relationship between facility characteristics and the prevalence of APM use. Second, the 44 NHs are not necessarily representative of all 300 NHs in Israel. Third, the study did not use data on residents’ characteristics as indications for the use of APMs. The strengths of this study are that this is the first description of APM use in Israeli NHs and that it provides some evidence of the impact of the NH facility’s characteristics on APM use.

In summary, this study shows that a greater use of APMs was associated with facility characteristics that generally indicate a lower quality of care. Shortage of staff, less recreational activity, and flawed equipment and daily living aids are all manifestations of poor quality of care. APMs are prescribed as chemical restraints in lieu of sufficient staff and appropriate activity for the residents of deficient facilities. Prescribing an antipsychotic therapy to a resident with no clinical indication for the therapy has been identified by the Centers for Medicare and Medicaid Services as a measure of poor quality of care [20].

In Israel, there are currently no formal guidelines on monitoring the use of APMs and we hope that this study will be instrumental in increasing the Ministry of Health’s supervision of the prescription of APMs nationwide. We believe that the inclusion of binding regulations on the prescription of APMs in addition to the development of skills for the care providers in non-pharmacological treatment of behavioral disorders will decrease APM use [21]. Recent data showed that the publication of the prevalence of APM use in NHs led to a remarkable decrease in the use of APMs elsewhere [1, 11, 22]. We therefore believe that making APM utilization data publically available in Israel will decrease the overall use of APMs in NHs.

## Conclusions

The wide variation in APM use that was found in NHs in the Tel Aviv district was associated with facility characteristics that undermine quality of care. The use of APMs as a measure of quality of care in Israeli NHs would probably lead to a decrease in their use and lower morbidity and mortality risks in NH residents.
